# Parameter Self-Adjusting Single-Mode Fiber Nutation Coupling Algorithm Based on Fuzzy Control

**DOI:** 10.3390/s25103051

**Published:** 2025-05-12

**Authors:** Yongkai Liu, Shuqiang Li, Furui Lv, Ximing Wang, Baogang Chen, Chenzi Guo, Kainan Yao, Jianli Wang

**Affiliations:** 1 Changchun Institute of Optics, Fine Mechanics and Physics, Chinese Academy of Sciences, Changchun 130033, China; lishuqiang23@mails.ucas.ac.cn (S.L.); lvfurui20@mails.ucas.ac.cn (F.L.); wangximing@ciomp.ac.cn (X.W.); cbg0813@163.com (B.C.); guocz@ciomp.ac.cn (C.G.); yaokainan001@126.com (K.Y.); wangjianli@ciomp.ac.cn (J.W.); 2 University of Chinese Academy of Sciences, Beijing 100049, China

**Keywords:** free-space optical communications, single-mode fiber, fiber nutation, parameter self-adjusting

## Abstract

The free-space optical communication fiber coupling system requires improved coupling efficiency through the fiber nutation algorithm. However, traditional fiber nutation algorithms suffer from issues such as fixed parameters and limited correction range. To address these challenges, a parameter self-adjusting single-mode fiber nutation coupling algorithm based on fuzzy control is proposed. Firstly, by leveraging the principles of fuzzy control and analyzing the influence of nutation parameters, a fuzzy controller with power and power change rate as input and nutation parameter as output is designed. Subsequently, we simulate and evaluate the static and dynamic performance of the fuzzy controller. The simulation results indicate that our algorithm outperforms traditional coupling algorithms in terms of coupling efficiency, accuracy, and speed. Finally, experimental results confirm the static and dynamic performance improvements of the parameter self-adjusting single-mode fiber nutation coupling algorithm based on fuzzy control. Specifically, under static conditions, the correction range of the proposed algorithm is approximately five times greater than that of the traditional algorithm. Under dynamic conditions, the disturbance suppression bandwidth increases to 8 Hz, nearly ten times greater than that of the traditional algorithm, significantly enhancing fiber nutation performance.

## 1. Introduction

Free-space optical communication (FSOC) is considered as an attractive candidate to provide high-speed wireless links, including satellite-to-ground ones [1]. Compared to traditional microwave communication, FSOC offers several advantages, including high data transmission rates, compact size, low energy consumption, enhanced confidentiality, and strong resistance to interference. Due to its exceptional communication performance, FSOC has emerged as a key development direction in the field of communications [2,3,4,5,6].

Ensuring efficient coupling of free-space optical beams into single-mode fiber (SMF) is a key challenge in high-performance FSOC systems [7,8]. In inter-satellite FSOC systems, due to the small core diameter of the SMF, the alignment precision of the beam is critically high, meaning that even the slightest errors, thermal effects, or mechanical disturbances can significantly reduce coupling efficiency. Furthermore, in satellite-to-ground FSOC systems, free-space optical beams are susceptible to atmospheric turbulence, which causes wavefront phase distortions, reducing transmission quality, increasing the bit error rate, and making the communication link unstable. To mitigate the wavefront distortions caused by atmospheric turbulence, adaptive optics (AO) technology has been widely applied. AO systems help to improve transmission quality by real-time detection and correction of wavefront aberrations. However, even after AO correction, factors such as temperature fluctuations and platform vibrations may still cause alignment misalignments, affecting coupling efficiency. Therefore, in both inter-satellite and satellite -to-ground FSOC systems, especially after AO systems correct for atmospheric turbulence, it is essential to introduce a fiber coupling system that can compensate for residual alignment errors, further improving fiber coupling efficiency.

Fiber coupling systems typically employ power–feedback sensors, which use the power coupled into the fiber as feedback. This approach provides several benefits, such as minimal sensitivity to environmental factors, low algorithmic complexity, and high reliability [9]. Currently, most fiber coupling systems utilize fast steering mirrors (FSM) as their actuation mechanism [10,11].

In 1990, Swanson et al. at MIT designed a system utilizing power feedback and a resonant fiber coupler to achieve the nutation function, with the system’s coupling efficiency reaching 63% [12]. Building on this, in 2020, Hu et al. introduced an adaptive stochastic parallel gradient descent (ASPGD) method to further enhance fiber coupling efficiency. By applying the ASPGD approach to model-free optimization problems, they successfully avoided local extrema and accelerated convergence. Simulations and experiments demonstrated that this method reduced iteration counts by 50%, while maintaining system stability [13]. In 2022, Bian et al. expanded on these advances by developing statistical expectation models to investigate the impact of optical system aberrations (OSA) and fiber misalignment errors on the coupling efficiency between free space and SMF under atmospheric turbulence. Their findings provide a solid theoretical foundation for the design and optimization of FSOC systems based on fiber coupling [14]. That same year, Zhang et al. introduced a fiber nutation-based mutual coupling method to achieve 10 Gbps transmission in near-ground quasi-static FSOC. By designing a coaxial laser terminal with a 50 mm aperture and using a single detector and actuator for beam acquisition, tracking, and nutation coupling, they significantly improved reception efficiency. Their method, validated through both indoor experiments and a 1 km field test, demonstrated an approximately 8 dB improvement in reception efficiency, enabling stable video transmission [15]. In 2024, Li et al. introduced a novel parameter-free fiber coupling method based on Gaussian approximation, which corrected non-coaxial errors and mitigated the effects of vibration in inter-satellite FSOC. Their swift and stable coupling technique operates autonomously, ensuring consistently high coupling efficiency. Experimental results indicated that this method effectively reduced bit error rates in high-vibration environments, ultimately achieving zero-bit errors under closed-loop control [16]. Most recently, in 2025, Pan et al. introduced an innovative FSOC scheme using a single adaptive fiber coupler to mitigate atmospheric turbulence and other disturbances at minimal cost. Experimental results in a 2 km urban environment demonstrated that this approach increased coupling power by up to 20% for the majority of the time. Additionally, after implementing closed-loop correction, the bit error rate decreased dramatically from 4.53 × 10^−4^ to 3.80 × 10^−8^, while also supporting successful video transmission. Furthermore, this scheme can be combined with coherent beam combining and spatial diversity techniques, providing a comprehensive solution to both tip/tilt aberrations and scintillation [17].

Currently, fiber coupling systems based on fiber nutation algorithms face a key challenge: the algorithm’s parameters remain fixed during the computation process, which limits the system’s ability to achieve both rapid convergence and high stability simultaneously.

This paper proposes a parameter self-adjusting SMF nutation coupling algorithm based on fuzzy control. The algorithm uses FSM as the actuating mechanism and adjusts its parameters in real-time based on the feedback of coupling power during the fiber nutation convergence process. Simulation and experimental results demonstrate that the coupling efficiency, accuracy, and speed of the proposed algorithm outperform those of traditional fiber nutation algorithms. Under conditions ensuring high coupling efficiency and accuracy, the self-adjusting fiber coupling algorithm corrects static errors approximately five times more effectively than the traditional approach, while achieving a disturbance suppression bandwidth up to ten times wider in dynamic scenarios.

The structure of this paper is as follows: Section 2 provides a brief overview of the relevant theories and presents simulations to evaluate the impact of nutation algorithm parameters, as well as analyzing the performance of the self-adjusting algorithm. Section 3 presents experimental results that validate the theoretical predictions. Finally, Section 4 concludes the paper, highlighting the superior performance of the parameter self-adjusting SMF nutation coupling algorithm based on fuzzy control.

## 2. Working Principle and Simulation Analysis

This section presents the theories and algorithms used in the simulations and experiments, including the principles of SMF coupling, the nutation algorithm, and the fuzzy control algorithm. Based on these theories and algorithms, the following analyses are carried out: First, the impact of nutation algorithm parameters on coupling performance is examined. Second, the parameter self-adjusting nutation algorithm based on fuzzy control is simulated and analyzed. A comparison with the traditional nutation algorithm [15,18,19] allows for the evaluation of both static and dynamic performance of the self-adjusting SMF nutation coupling algorithm.

### 2.1. SMF Coupling Principle

**Figure 1:**
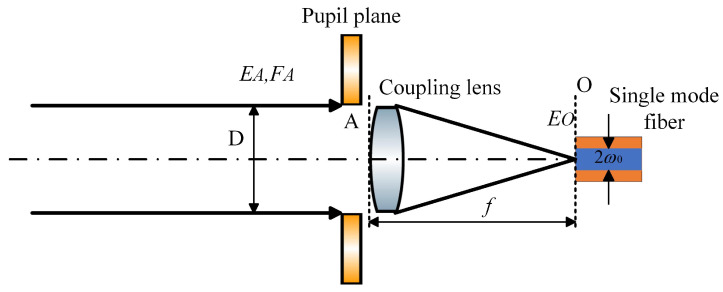
SMF coupling principle.

After the optical beam is transmitted over long distances through the FSOC system, the laser beam at the receiver end can be approximated as a plane wave. When this plane wave passes through a lens and converges, it forms an Airy spot at the focal plane. To maximize the optical power coupled into the SMF, the center of the Airy spot must coincide with the center of the SMF mode field. As shown in Figure 1, $E_{A}$ represents the plane optical field on the entrance pupil surface A, and $E_{O}$ is the field formed at the focal plane O after the plane wave passes through the lens. Here, D is the diameter of the lens, $\omega_{0}$ is the radius of the SMF mode field, and f is the focal length of the lens.

The coupling efficiency of space light into a SMF is defined as the ratio of the optical power of spot $E_{O}$ on the focal plane O to the optical power entering the SMF. Due to the complexity involved in fitting the wavefront phase of $E_{A}$ using Zernike polynomials, coupling efficiency can alternatively be computed on the entrance pupil surface A using Parseval’s theorem, as referenced in [20].

(1) $$\eta =\frac{{\left|\iint {E_{A}}^{*}(\rho ,\theta )F_{A}(\rho ,\theta )\rho d\rho d\theta \right|}^{2}}{\iint {\left|E_{A}(\rho ,\theta )\right|}^{2}\rho d\rho d\theta \cdot \iint {\left|F_{A}(\rho ,\theta )\right|}^{2}\rho d\rho d\theta }$$

where (ρ, θ) is the polar coordinate of any point on the focal plane O, and $F_{A}$ is the backward transmission mode field formed at the pupil plane after backward transmission of the SMF mode field. The expression is:

(2) $$F_{A}(\rho ,\theta )=\sqrt{\frac{2}{\pi {\omega_{a}}^{2}}}\exp(-\frac{\rho^{2}}{{\omega_{a}}^{2}})$$

where $\omega_{a}$ is the radius of the backward transmission mode field, calculated as: $\omega_{a}=\;\lambda \;f/\pi \omega_{0}$, the incident light field is $E_{A}={\;E}_{S}\;\exp\;[-j\;\phi \;(\rho ,\;\theta )]$; in Equation (1), if the integration range is the full effective aperture of the incident pupil plane, then there is:

(3) $$\begin{array}{ll} \eta & =\frac{{\left|\iint {E_{A}}^{*}(\rho ,\theta )F_{A}(\rho ,\theta )\rho d\rho d\theta \right|}^{2}}{\iint {\left|E_{A}(\rho ,\theta )\right|}^{2}\rho d\rho d\theta \cdot \iint {\left|F_{A}(\rho ,\theta )\right|}^{2}\rho d\rho d\theta }\\ & ={\left|\iint \exp\left[-j\phi (\rho ,\theta )\right]\cdot \sqrt{\frac{2}{\pi {\omega_{a}}^{2}}}\exp(-\frac{\rho^{2}}{{\omega a}^{2}})\rho d\rho d\theta \right|}^{2}\\ & =\frac{2}{\pi^{2}\rho^{2}{\omega_{a}}^{2}}({\alpha_{r}}^{2}+{\alpha_{j}}^{2}) \end{array}$$

where

(4) $$\alpha_{r}=\iint \exp(-\frac{\rho^{2}}{{\omega_{a}}^{2}})\cos\left[\phi (\rho ,\theta )\right]\rho d\rho d\theta$$

(5) $$\alpha_{j}=\iint \exp(-\frac{\rho^{2}}{{\omega_{a}}^{2}})\sin\left[\phi (\rho ,\theta )\right]\rho d\rho d\theta$$

According to Equations (3)–(5), the coupling efficiency of space light to SMF under different wavefront phases can be obtained.

### 2.2. Fiber Nutation Algorithm

In the FSOC systems, the fiber nutation algorithm is a widely used fundamental method for fiber coupling. Firstly, FSM is used to make the spot on the focal plane undergo circular motion with a certain radius. The system identifies the position of maximum power through power sampling, records it, and adjusts the scanning center to this position using FSM. After multiple scans, the system eventually converges to a globally optimal position, thereby achieving maximum coupling efficiency. The detailed process is outlined as follows:

(1) The algorithm initializes parameter setting, and sets the scanning center $\left(X_{0},Y_{0}\right)$, the nutation radius r, nutation step length d, and the number of sampling points n per revolution.
(2) Output the control quantity: calculate the control quantity $\left({X_{0}}^{\prime },\;{Y_{0}}^{\prime }\right)$, where:
  (6) $$\begin{array}{cc} \left\{\begin{array}{l} {X^{\prime }}_{0}=X_{0}+rcos\left(2\pi m/n\right)\\ {Y^{\prime }}_{0}=Y_{0}+r\sin\left(2\pi m/n\right) \end{array}\right. & m=0,1,\ldots ,n-1 \end{array}$$

The control quantity and nutation radius r have the same dimension. The control quantity for the scanning cycle is output, each position is sampled, and the position where the maximum coupling efficiency is reached $\left(X_{\mathrm{best}},{\;Y}_{\mathrm{best}}\right)$ and the sampling number I at the maximum optical power are recorded.

**Figure 2:**
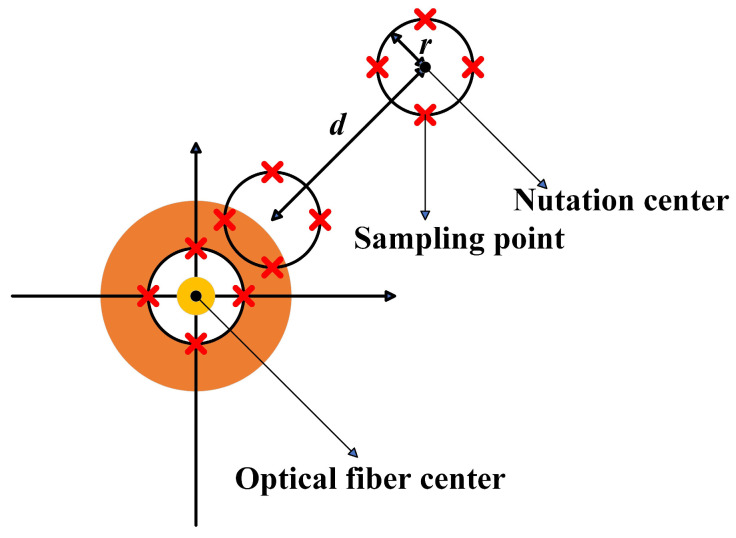
Schematic diagram of fiber nutation.

(3) Move the scanning center: move the scanning center in the direction of the maximum coupling efficiency according to the set nutation step length d, that is,
  (7) $$\left\{\begin{array}{l} X_{0}=X_{0}+dcos\left(2\pi \left(I-1\right)/n\right)\\ Y_{0}=Y_{0}+dsin\left(2\pi \left(I-1\right)/n\right) \end{array}\right.$$
(4) Repeat steps (2) to (3). The above process can be represented by Figure 2.

### 2.3. Analysis of the Influence of Nutation Parameters on Coupling Performance

The calculation process of the SMF nutation algorithm is simulated, and the impact of selecting different nutation parameters on coupling performance is analyzed. Understanding how nutation parameters affect system coupling performance is essential for designing a self-adjusting algorithm. Based on the practical requirements of SMF coupling, the following performance indicators are proposed:

(1) Coupling speed: the time required for the SMF coupling power to reach its maximum value from the initial value after the algorithm is activated.
(2) Coupling stability: the fluctuation of coupling power after steady-state coupling is achieved, described by the steady-state standard deviation, denoted as σ.
(3) Coupling efficiency: the steady-state coupling power divided by the input power, with the input power set to 1 in the simulation.

In the absence of external disturbances, the primary attentions are given to the rapidity of coupling and the value of coupling power. Achieving quick coupling of the Airy spot into the fiber is crucial in a static environment. When external disturbances are introduced, the main concerns are the stability of the coupling and the value of the coupling power. Because the external disturbances have been applied, the coupling speed in this index cannot be judged; for the system, it is more important to value its anti-interference ability.

By calculating with the above parameters (in Table 1), the diameter of the Airy spot transmitted to the end face of the SMF is approximately 18.9 μm, and the energy of the Airy spot is mainly concentrated in the central bright region (i.e., at the 1/e^2^ power point, corresponding to a 13.5% power loss). We calculated the radius at this point to be $r_{airy}=2.584\lambda f/\pi D$ = 6.37 μm, and the diameter is 12.74 μm, while the mode field diameter of the SMF $\omega_{0}\;$ is 10.4 μm, the radius of the backward transmission mode field $\omega_{a}=\;\lambda \;f/\pi \omega_{0}$ = 4.74 mm, D/2 $\omega_{a}$ = 1.0548. the coupling parameter $\beta =D/2\omega_{a}=\pi D\omega_{0}/2\lambda f=1.0548$, Based on actual application needs, the initial deviation between the center of the SMF and the center of the Airy spot is set as 5 μm.

**Figure 3:**
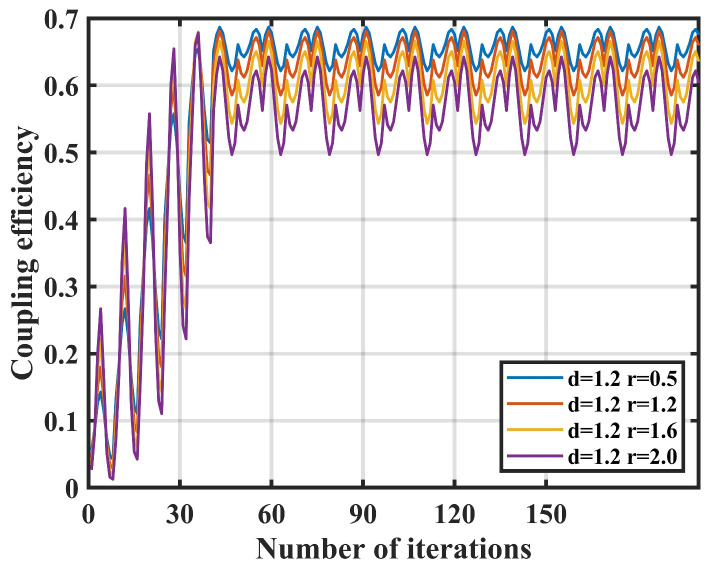
Effect of nutation radius *r* on coupling efficiency.

Firstly, the impact of nutation radius on coupling performance is simulated. The nutation step length d is fixed at 1.2 μm, the number of sampling points n is fixed at 4, and the nutation radius r varies to 0.8 μm, 1.2 μm, 1.6 μm, and 2.0 μm for simulation. The simulation results are shown in Figure 3.

When the fixed nutation step length d and the number of sampling points n remain constant, after approximately 48 iterations, all data sets of data reach a steady state, with coupling efficiency above 0.5. Thus, varying the nutation radius r has little impact on the coupling speed of the system.

With the increase in nutation radius r, the oscillation amplitude of the system at steady state significantly rises. When the nutation radius r is 2.0 μm, the steady-state oscillation of the system reaches the maximum. Therefore, adjusting the nutation radius r has a profound effect on the system’s coupling stability.

**Figure 4:**
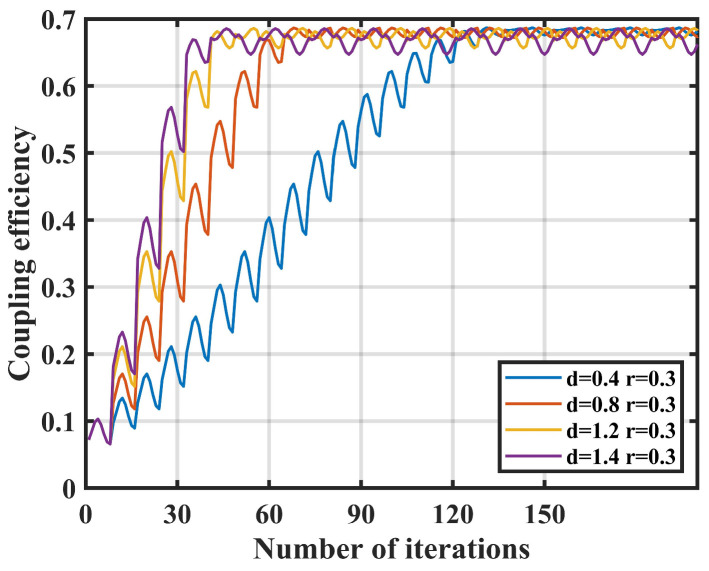
Effect of nutation step d on coupling efficiency.

Then, the effect of nutation step length d  on coupling performance is simulated. The nutation radius r is fixed at 0.3 μm, the sampling number n is fixed at 4, and simulations are conducted with nutation step lengths d of 0.4 μm, 0.8 μm, 1.2 μm, and 1.4 μm. The results of coupling efficiency and the number of nutation are shown in Figure 4.

When the nutation radius r and the number of sampling points n remain constant, the power fluctuations in the steady state for the compared data sets are not much different, with the coupling efficiency is greater than 0.6. Therefore, changing the nutation step length d has a smaller impact on the coupling stability of the system.

When the nutation step length d is increased, the number of iterations required for the system to reach a steady state is significantly reduced. When nutation step length d = 1.4 μm, only 47 iterations are needed to achieve the steady state; when nutation step length d = 0.4 μm, the number of iterations to reach the steady state is 125. Therefore, adjusting nutation step length d has a great influence on the coupling speed.

**Figure 5:**
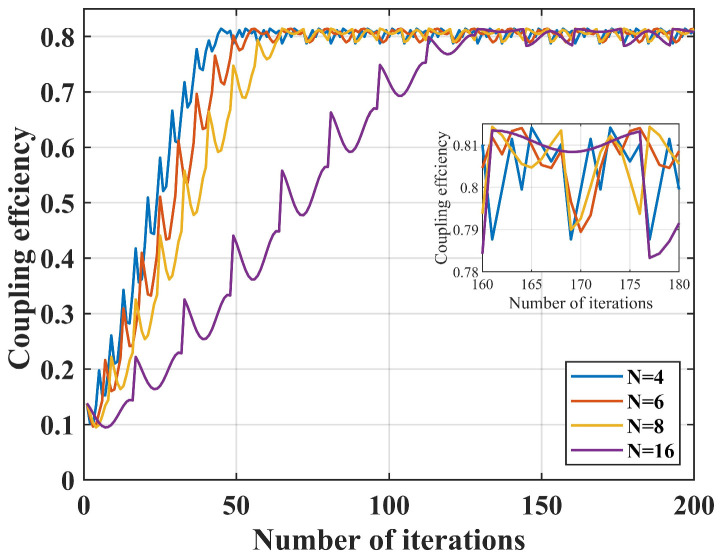
Effect of *n* on coupling efficiency.

Finally, the impact of the number of sampling points on coupling performance is simulated. Given that the deviation of 9 μm between the center of the SMF and the center of the Airy spot, and the nutation radius r is fixed at 0.3 μm, the nutation step length d is fixed at 0.8 μm, and the number of switching sampling points n is 4, 6, 8, 16, the simulation results are shown in Figure 5.

When n = 4, the number of iterations required to reach a steady state is the smallest, but the rate of change in power is the largest in unit time, resulting in poorer coupling stability. Conversely, when n = 16, the number of iterations required to reach steady state is the greatest, but the rate of change in power is minimal both during the coupling process and after reaching steady state. Therefore, when the nutation radius r and the nutation step length d remain constant, changing the number of sampling points *n* also affects the coupling speed and stability to some extent.

In the process of implementing the fiber nutation algorithm, when the nutation radius r and nutation step length d are small, the convergence speed decreases, but the convergence stability improves. Conversely, when r and d are large, the convergence speed increases, but the convergence stability decreases. Additionally, the number of sampling points n per cycle also influences both the convergence speed and stability. Therefore, the nutation coupling system must select appropriate nutation parameters based on various factors. The SMF nutation coupling algorithm based on fuzzy control can adaptively adjust the nutation parameters during the algorithm’s execution to ensure both fast convergence and high steady-state accuracy. Based on the analysis of the simulation results above, the parameter self-adjustment algorithm should include the following processes:

(1) In the initial stage of fiber nutation, the laser coupling power is low, and the power change rate is small. A large nutation radius r combined with a large nutation step length d is used to quickly position the initial coupling location, while avoiding the failure to capture the Airy spot due to jitter.

(2) In the middle stage of fiber nutation, the laser coupling power reaches a medium level, and the power change rate increases. A medium nutation radius r combined with a medium nutation step length d is used to rapidly move the Airy spot to the center of the SMF, avoiding the impact of local optimization.

(3) In the steady-state stage of fiber nutation, the laser coupling power is high, and the power change rate stabilizes. A small nutation radius r combined with a small nutation step length d is used to enhance the steady-state accuracy of the system and maintain its continuous correction capability.

### 2.4. Fuzzy Control Algorithm

**Figure 6:**
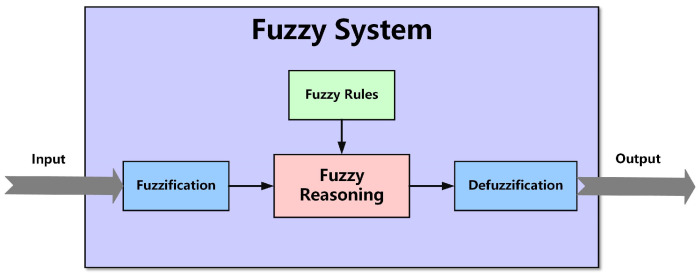
Fuzzy control system structure.

Fuzzy control is a control strategy based on the principles of fuzzy logic, designed to address system issues that are challenging to resolve with traditional control methods due to complexity, nonlinearity, uncertainty, or fuzziness [21,22,23]. Therefore, this paper combines the fuzzy control algorithm with the fiber nutation algorithm to achieve parameter self-adjustment in the fiber nutation process. Figure 6 illustrates a typical fuzzy control system structure.

A fuzzy control system typically consists of three fundamental components [24,25]:

(1) Fuzzification: the primary objective of this step is to convert system input values into fuzzy set values. These fuzzy sets are usually represented by linguistic variables (such as low, medium, and high), which describe the different degrees or ranges of the input variable.

Suppose that the exact input value of an input variable $x_{i}$ is $\;x_{i}^{*}$, we need to compute the membership of that input value in all fuzzy sets of that variable.

The membership function is defined as:

(8) $$\mu_{A_{i}^{k}}(x_{i}):X_{i}\to [0,1]$$

where $X_{i}$ is the domain of the input variable ${\;x}_{i}$, and μ is the membership function. For the input value $x_{i}^{*}$, calculate

(9) $$\mu_{A_{i}^{k}}(x_{i}^{*}),\;\;\;k=1,2,\ldots ,K_{i}$$

where $K_{i}$ is the number of language-valued classes of the input variable ${\;x}_{i}$.

(2) Fuzzy reasoning: after fuzzification, the system applies a set of fuzzy rules to make decisions. These rules define the relationship between inputs and outputs. The fuzzy control algorithm utilizes these rules to determine the corresponding output actions based on specific input combinations. Calculate the membership of a rule premise.

For Rule *j*:$\;R_{j}$: if $x_{1}$ is a $\;A_{1}^{j}$ and $x_{1}\;$ is a $A_{2}^{j}$, $x_{n}$ is a $\;A_{n}^{j}$, then *y* is ${\;B}^{j}$. First, calculate the membership of all the premises:

(10) $$\mu_{A_{i}^{j}}(x_{i}^{*}),\;\;\;i=1,2,\ldots ,n$$

Then, the fuzzy logic operator “And” is used to connect the premises using the minimum operator:

(11) $$\alpha_{j}=\min_{1\leq i\leq n}\mu_{A_{i}^{j}}(x_{i}^{*})$$

where $\alpha_{j}$ is the trigger strength (or confidence level) of Rule *j*. Adjustment rule conclusion is a fuzzy set. According to the Mamdani method, the conclusion of a rule is a fuzzy set ${\;B}^{j}$ that is “Cut” or “Scaled” according to the membership ${\;\alpha }_{j}$ of the premise. The most common method is the cut method:

(12) $$\mu_{B^{j}}^{\prime }(y)=\min(a_{j},\mu_{B^{j}}(y))$$

All the output fuzzy sets obtained by rule reasoning are combined into a single-output fuzzy set.

(13) $$\mu_{B^{1}}^{\prime }(y),\mu_{B^{2}}^{\prime }(y),\ldots ,\mu_{B^{M}}^{\prime }(y)$$

The aggregation operator joins the output fuzzy sets of all rules with an “OR”, usually using the max operator:

(14) $$\mu_{B}(y)=\max_{1\leq j\leq M}\mu_{B^{j}}^{\prime }(y)$$

(3) Defuzzification: this step converts the outputs from the fuzzy inference stage into precise control action values. The defuzzification process generates the final non-fuzzy output value, which serves as the actual control input for the fiber nutation algorithm by considering all possible output fuzzy sets and their respective membership degrees. The formula is:

(15) $$y^{*}=\frac{\int y\mu_{B}(y)dy}{\int \mu_{B}(y)dy}$$

where the integral interval is the domain of the output variable *y*. Convert the fuzzy output set *B* to a precise numerical output.

### 2.5. Fuzzy Controller Design

Due to the fact that the fiber nutation algorithm only utilizes real-time coupling power as feedback, it can obtain real-time coupling power P and power change rate Pt, the power change rate Pt is calculated by coupling the power P change in real time. According to the characteristics of nutation algorithm, we define the power change rate as the difference between the average power of the current scanning cycle and the average power of the previous scanning cycle, that is:

(16) $$Pt=P_{mean}(T)-P_{mean}(T-1)$$

where $\;P_{mean}(T)$ represents the average coupling power of the current scanning cycle, and $P_{mean}(T-1)$ represents the average coupling power of the previous scanning cycle. This approach is compatible with the fuzzy control self-adjusting algorithm, which adjusts parameters based on the deviation E and deviation change rate Ec [26,27]. Consequently, the design parameters of the fuzzy controller include coupling power P, power change rate Pt, nutation radius r, nutation step length d, and the number of sampling points n.

Based on the aforementioned lemma and a statistical analysis of real-time coupling power data collected during the single-mode fiber coupling process, the fuzzy domain of the coupling power P was set to [0, 0.7].

After analyzing the power change rate data under various operating conditions and referring to Equation (16), the fuzzy domain for the power change rate, denoted as Pt, was determined to be in the range of [−0.35, 0.4]. Additionally, multiple combinations of nutation step length d and nutation radius r were selected for extensive simulations to evaluate the system’s performance under both static and dynamic conditions. In the static scenario, the rise time and steady-state accuracy were assessed, while in the dynamic scenario, the results under added disturbances were analyzed. Considering the trade-offs among rise time, steady-state accuracy, and disturbance resistance, the fuzzy domains for d and r were ultimately determined as (0, 2.5] and (0, 1.0], respectively.

The sampling number n also plays a critical role in the fiber coupling system, which relies solely on coupling power as feedback. To ensure that the nutation algorithm can move in the correct convergence direction, the minimum sampling number n was set to 4, avoiding inaccuracies in determining the convergence path caused by insufficient sampling. Furthermore, during the later stages of adjustment, as power variations become more gradual, increasing the sampling number improves the precision of direction determination and enhances decision-making accuracy. However, an excessively large sampling number can reduce the system’s response speed, thereby diminishing its ability to suppress errors. Balancing the trade-offs between system accuracy and dynamic performance, the fuzzy domain for the sampling number n was ultimately set to [4,8].

Then, the fuzzy domains of the above parameters are divided, respectively, to obtain the fuzzy sets for these five parameters, represented as follows: the fuzzy set of coupling power is P ={S,M,L}, the fuzzy set of power change rate is Pt ={S,L}, the fuzzy set of nutation radius and the fuzzy set of nutation step length are r, d ={VS,S,M,L,XL}, and the fuzzy set of the number of sampling points is n ={F,H}, where vs. denotes very small, S denotes small, M denotes medium, L denotes large, XL denotes very large, F denotes few, and H denotes many.

Finally, membership functions for the above parameters are constructed based on fuzzy domains and fuzzy sets, respectively. Typical membership functions in the design of fuzzy control systems include Gaussian, S-shaped, trapezoidal, and triangular functions, each suitable for different application scenarios. The shape of the triangular membership function is mainly described by the slope parameter, which is not only easy to calculate, but also easy to adjust according to the change in the dynamic conditions of the system. Therefore, this paper employs triangular membership functions to construct the fuzzy control strategy.

**Figure 7:**
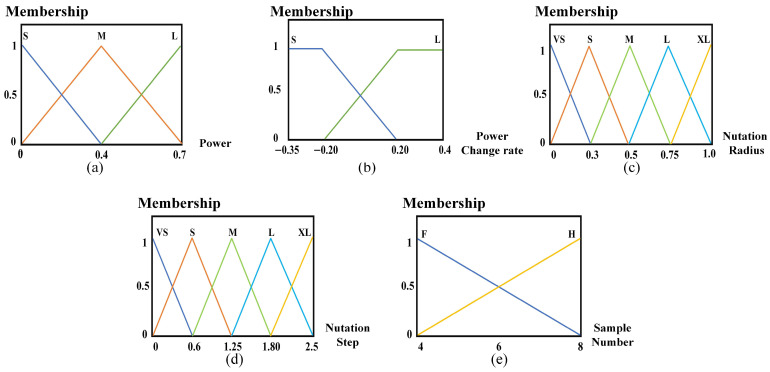
The membership function of each parameter: (**a**) coupling power P membership function; (**b**) coupling power change rate Pt membership function; (**c**) nutation radius *r* membership function; (**d**) nutation step d membership function; (**e**) sample number *n* membership function.

The membership function diagram of the constructed coupling power, power change rate, nutation radius, nutation step length, and the number of sampling points are shown in Figure 7.

Taking Figure 7a as an example, the graph represents the relationship between coupling power and membership degree. When the coupling power is 0, the membership degree of S is 1, while that of M and L is 0. As the coupling power gradually increases, the membership degree of S gradually decreases, that of M gradually increases, and that of L remains 0. Thus, the probability of selecting M in the corresponding coupling power calculation gradually increases. When the coupling power is 0.4, the membership degree of S is 0, that of M is 1, and that of L is 0, indicating that a coupling power of 0.4 corresponds entirely to M. As the coupling power continues to increase, the membership degree of S remains 0, that of M gradually decreases, and that of L gradually increases, thus increasing the probability of selecting L in the corresponding coupling power calculation. Similarly, the membership functions of other parameters can also be expressed and their corresponding relationships.

Fuzzy inference is the core process of a fuzzy control system, which involves analyzing and judging the input variables after fuzzification based on a set of predefined fuzzy rules to determine the corresponding output control actions. According to the conclusions in Section 2.3, the fuzzy rules are formulated as shown in Table 2.

We use the center of maximum membership method to transform the fuzzy quantities into the output results of the membership function, and then convert the fuzzy quantities generated by the fuzzy control system into specific numerical outputs.

Taking the nutation radius r as an example, the output nutation radius r is:

(17) $$r=\frac{\int_{0}^{1}r\cdot \mu (r)\cdot dr}{\int_{0}^{1}\mu (r)\cdot dr}$$

In the above formula, μ(r) is the membership degree of r, and the calculation method is given by the following formula:

(18) $$\mu (r)=\max\{{\mu (r)}_{VS},\;{\mu (r)}_{S},\;{\mu (r)}_{M},\;{\mu (r)}_{L},\;{\mu (r)}_{XL}\}$$

(19) $${\mu (r)}_{VS}=\left\{\begin{array}{ll} 0 & r_{0}\leq 0\\ \frac{0.3-r_{0}}{0.3} & 0\leq r_{0}<0.3\\ 0 & r_{0}\geq 0.3 \end{array}\right.$$

(20) $${\mu (r)}_{S}=\left\{\begin{array}{ll} 0 & r_{0}\leq 0\\ \frac{r_{0}}{0.3} & 0\leq r_{0}<0.3\\ \frac{0.5-r_{0}}{0.2} & 0.3\leq r_{0}<0.5\\ 0 & r_{0}\geq 0.5 \end{array}\right.$$

(21) $${\mu (r)}_{M}=\left\{\begin{array}{ll} 0 & r_{0}\leq 0.3\\ \frac{r_{0}-0.3}{0.2} & 0.3\leq r_{0}<0.5\\ \frac{0.75-r_{0}}{0.25} & 0.5\leq r_{0}<0.75\\ 0 & r_{0}\geq 0.75 \end{array}\right.$$

(22) $${\mu (r)}_{L}=\left\{\begin{array}{ll} 0 & r_{0}\leq 0.5\\ \frac{r_{0}-0.5}{0.25} & 0.5\leq r_{0}<0.75\\ \frac{1.0-r_{0}}{0.25} & 0.75\leq r_{0}<1.0\\ 0 & r_{0}\geq 1.0 \end{array}\right.$$

(23) $${\mu (r)}_{XL}=\left\{\begin{array}{ll} 0 & r_{0}\leq 0.75\\ \frac{r_{0}-0.75}{0.25} & 0.75\leq r_{0}<1.0\\ 0 & r_{0}\geq 1.0 \end{array}\right.$$

where r represents the new nutation radius, $r_{0}$ represents the radius mapping value, ${\mu (r)}_{\mathrm{vs}}$ represents the membership degree of the radius corresponding to vs. in the fuzzy set, ${\mu (r)}_{s}$ represents the membership degree corresponding to S in the fuzzy set, ${\mu (r)}_{M}$ represents the membership degree corresponding to M in the fuzzy set, ${\mu (r)}_{L}$ represents the membership degree corresponding to L in the fuzzy set, and ${\mu (r)}_{XL}$ represents the membership degree corresponding to XL in the fuzzy set.

The calculation process of the fuzzy controller is illustrated through the following example: When the coupling power P =0.5 and the power change rate Pt=0.3, the determination of the current nutation radius r is as follows:

(1) Determine membership degrees: for P =0.5, according to Figure 7a, the corresponding membership degrees are M (2/3) and L (1/3). For  Pt =0.3, according to Figure 7b, the membership degree is  L (1).

(2) Lookup and compute membership relationships: using Table 2, according to Figure 7c, the membership degrees corresponding to the nutation radius determined to be  M (2/3) and S (1/3).

(3) Convert fuzzy quantities into outputs: the center of maximum membership method is employed to convert the fuzzy quantities into outputs. For the nutation radius r with the fuzzy quantity M (2/3), substituting into Equation (21) yields two possible output values: r =0.33 and r=0.58.

(4) Calculate the average: to obtain the final output, the two values are averaged, resulting in a new nutation radius,  r =0.45.

**Figure 8:**
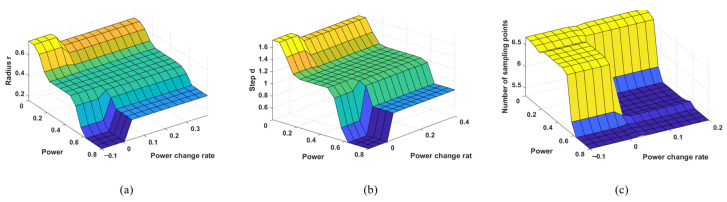
Controller inference conclusion stereogram: (**a**) Fuzzy Controller 1; (**b**) Fuzzy Controller 2; (**c**) Fuzzy Controller 3.

Based on the above process, fuzzy rules and membership functions are set in the simulation software toolbox to obtain the following fuzzy controllers: Fuzzy Controller 1, which uses coupling power P  and power change rate  Pt  as inputs and nutation radius r as output; Fuzzy Controller 2, which uses coupling power P and power change rate Pt as inputs and nutation step length d  as output; and Fuzzy Controller 3, which uses coupling power P and power change rate Pt as inputs and the number of sampling points n as output. After setting these up in the simulation software, a three-dimensional plot of the fuzzy controller inference conclusions can be obtained, as shown in Figure 8.

After completing the above process, the entire design workflow of the parameter self-adjusting nutation algorithm can be accomplished according to the existing fuzzy controllers.

**Figure 9:**
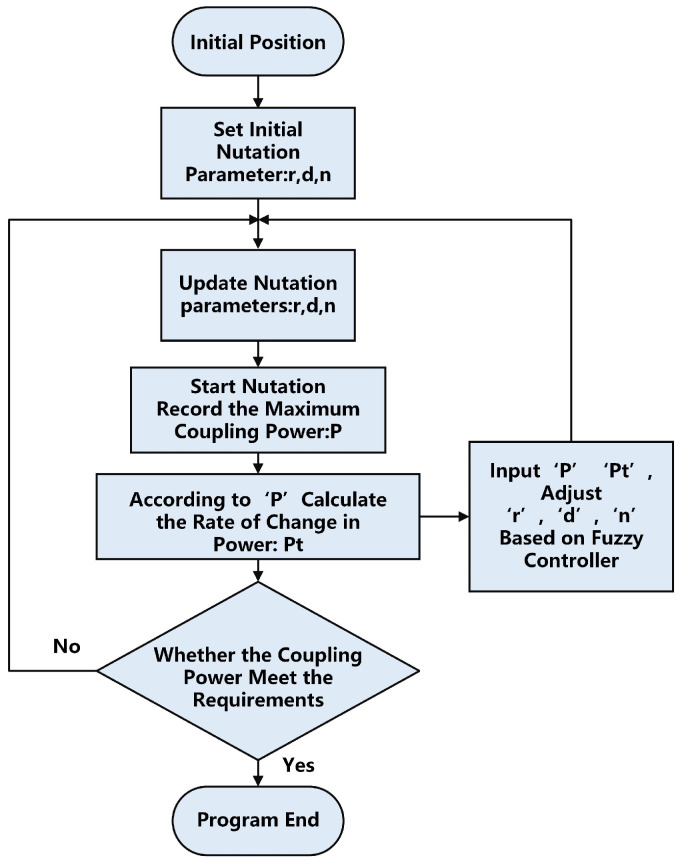
Flowchart of the self-adjusting algorithm.

Firstly, based on the given initial nutation parameters, a single cycle scan is performed, and the maximum coupling power value for that cycle is recorded. Then, using the current power value and the calculated rate of change in power, the input parameters for the fuzzy controller are determined. The fuzzy controller subsequently computes the defuzzified new nutation parameters for the next scan iteration. This process continues until the coupling power reaches the desired level or the maximum value is achieved. The corresponding algorithm flowchart is shown in Figure 9.

### 2.6. Simulation and Analysis of Nutation Coupling Algorithm for SMF with Parameter Self-Adjusting Based on Fuzzy Control

**Figure 10:**
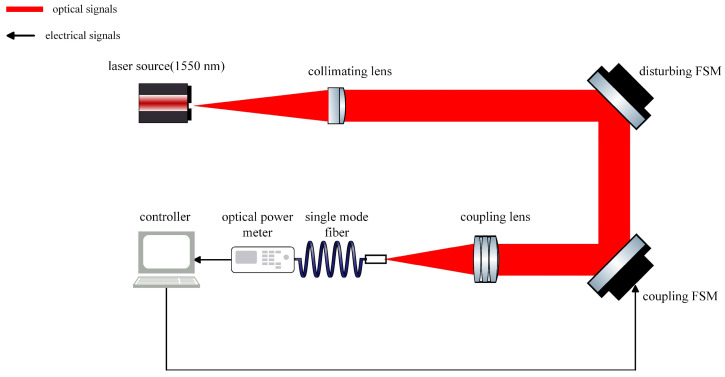
Schematic diagram of simulation experiment.

The schematic diagram of the simulation experiment based on the FSOC SMF coupling system is shown in Figure 10.

**Figure 11:**
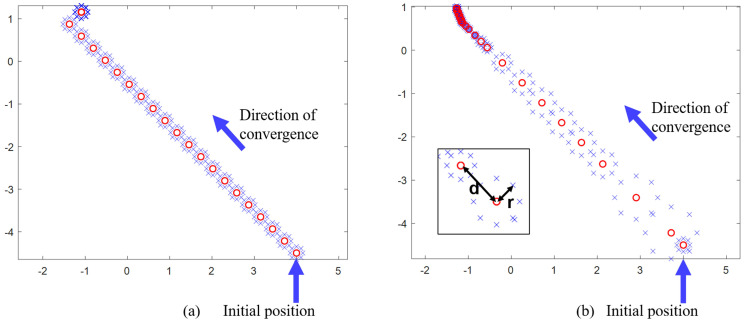
Schematic diagram of the nutation path in the simulation process: (**a**) traditional fiber nutation; (**b**) parameter self-adjusting nutation.

After the laser-generated beam is aligned, it first passes through a disturbed FSM, which introduces external disturbances with specific amplitudes and frequencies. The beam is then focused and coupled into an SMF using a coupling lens. The coupling FSM is controlled by a controller that performs parameter self-adjusting nutation scanning to compensate for the disturbances. The optical power meter collects power values as feedback, which are then input into the controller. The controller calculates the nutation parameters and sends the control variables back to the coupled FSM. The system parameters used in the simulation are listed in Table 1. The schematic diagram of the nutation path in the simulation process of both the traditional fiber nutation and the parameter self-adjusting nutation algorithm is shown in Figure 11.

Firstly, a simulation analysis of the coupling performance of the parameter self-adjusting algorithm in a static state is conducted. The disturbed FSM remains stationary, while the coupling FSM is given an initial lateral offset. The algorithm controls the coupling FSM to move the Airy spot to the position of maximum coupling power. In the simulation, the input power is set to a fixed value, and the coupling power is represented by the coupling efficiency.

**Figure 12:**
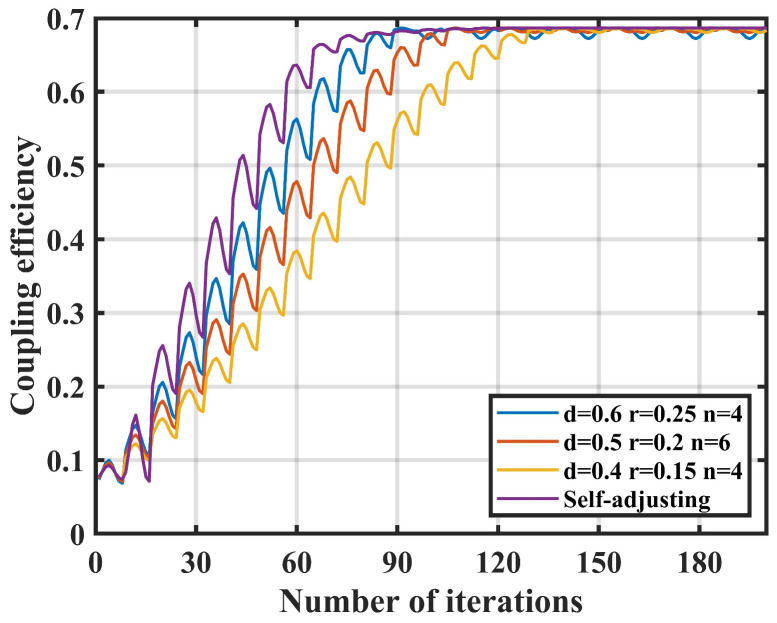
Static condition coupling performance comparison.

Three sets of traditional fiber nutation controllers and parameter self-adjusting controllers are selected for comparison. The comparison includes whether the iteration has reached the maximum coupling power position, the number of iterations, and the calculation of the average coupling efficiency (η) and the standard deviation (σ) after steady-state. The results are presented in Table 3 and Figure 12.

Based on the simulation results, it is clear that the parameter self-adjusting nutation coupling algorithm based on fuzzy control significantly outperforms the traditional nutation coupling algorithm. The self-adjusting algorithm increases the average coupling efficiency to 0.683, achieves the lowest standard deviation of 0.0001, and results in an average steady-state iteration count of 82.

Next, a simulation analysis is conducted to evaluate the coupling performance of the parameter self-adjusting algorithm under dynamic disturbances. To simulate the dynamic disturbances encountered in practical applications, a sinusoidal waveform disturbance with a peak-to-peak value (PV) of 20 μrad is introduced. This allows for a more intuitive observation and analysis of the coupling performance and disturbance suppression ability of both the parameter self-adjusting nutation coupling algorithm and the traditional nutation algorithm under periodic variations. The evaluation metrics for this simulation include coupling efficiency and system stability.

In this paper, eight sets of traditional fiber nutation algorithms and parameter self-adjusting nutation algorithms are selected to compare their coupling effects under disturbed conditions. The parameters and results of the fiber nutation algorithms, including PV, root mean square (RMS), and σ, are shown in Table 4.

**Figure 13:**
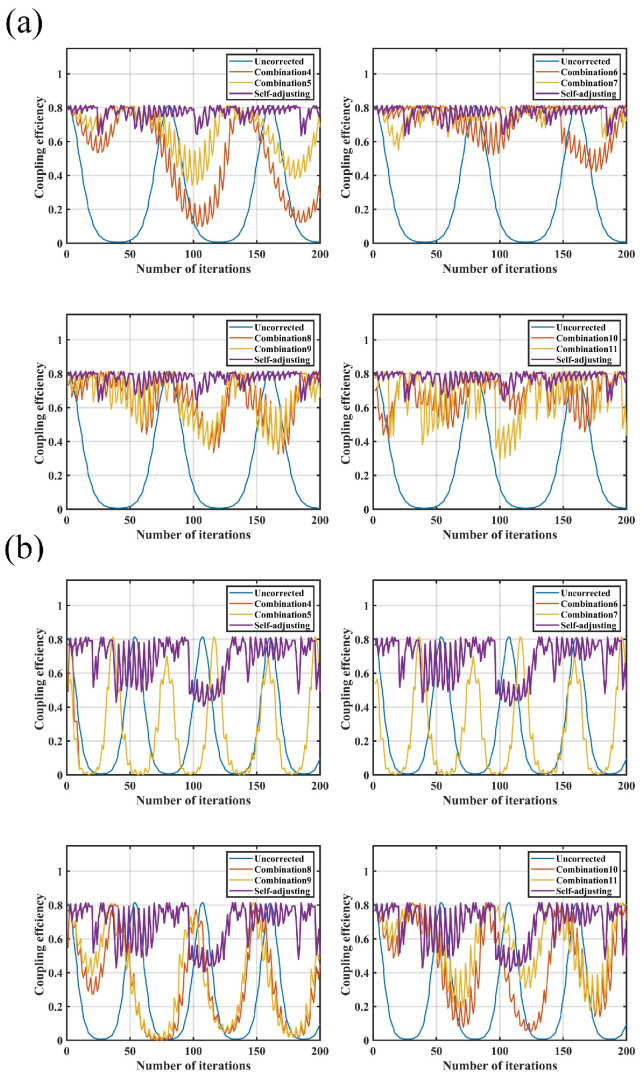
Comparison of coupling performance after adding disturbance: (**a**) comparison of coupling performance under 0.5 Hz disturbance; (**b**) comparison of coupling performance under 1.0 Hz disturbance.

After introducing external disturbances of 0.5 Hz and 1.0 Hz, the comparison of coupling performance between the parameter self-adjusting nutation coupling algorithm and the traditional algorithm is shown in Figure 13.

The PV of coupling efficiency in the uncorrected state is 0.807. In comparison, under a 0.5 Hz disturbance, the parameter self-adjusting nutation algorithm demonstrates superior correction performance over the traditional nutation algorithm. When exposed to a 1.0 Hz disturbance, only a few parameter combinations of the traditional nutation algorithm show some effect, while the parameter self-adjusting nutation algorithm effectively suppresses external disturbances within a narrower range. Therefore, the parameter self-adjusting coupling algorithm exhibits better correction capability.

**Figure 14:**
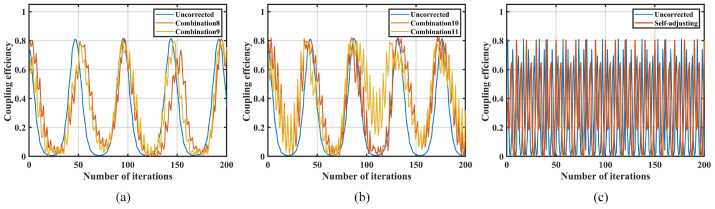
Comparison of perturbation bandwidths of different combinations: (**a**) coupling performance of combinations 8 and 9 at 1.1 Hz interference; (**b**) coupling performance of combinations 10 and 11 at 1.2 Hz interference; (**c**) coupling performance of self-adjusting at 10 Hz interference.

The frequency of external disturbances is gradually increased to determine the disturbance suppression bandwidth (the definition of suppression bandwidth is the frequency of the disturbance signal at which the fluctuation in the corrected optical power is close to the amplitude of variation observed when the system is uncorrected) of the parameter self-adjusting nutation coupling algorithm. This bandwidth is then compared with the disturbance suppression bandwidth of the traditional fiber nutation algorithm for parameter combinations 4 through to 11, as shown in Figure 14 and Table 5.

The disturbance suppression bandwidth of the parameter self-adjusting nutation coupling algorithm reaches 10 Hz, while the traditional fiber nutation algorithm achieves a maximum bandwidth of only 1.2 Hz. In comparison to the traditional fiber nutation algorithm, the parameter self-adjusting nutation coupling algorithm significantly enhances the anti-interference capability of the SMF coupling system, while also maintaining coupling accuracy and stability.

### 2.7. Summary

The parameter self-adjusting SMF nutation coupling algorithm based on fuzzy control improves the average coupling efficiency to 0.687, with a standard deviation of 0.0001, and achieves an average steady-state iteration count of 81. This significantly enhances the adjustment range, speed, and steady-state accuracy under static conditions. Under dynamic conditions, the disturbance suppression bandwidth reaches 10 Hz, which is approximately 10 times higher than that of the traditional fiber nutation algorithm 

## 3. Experiment

**Figure 15:**
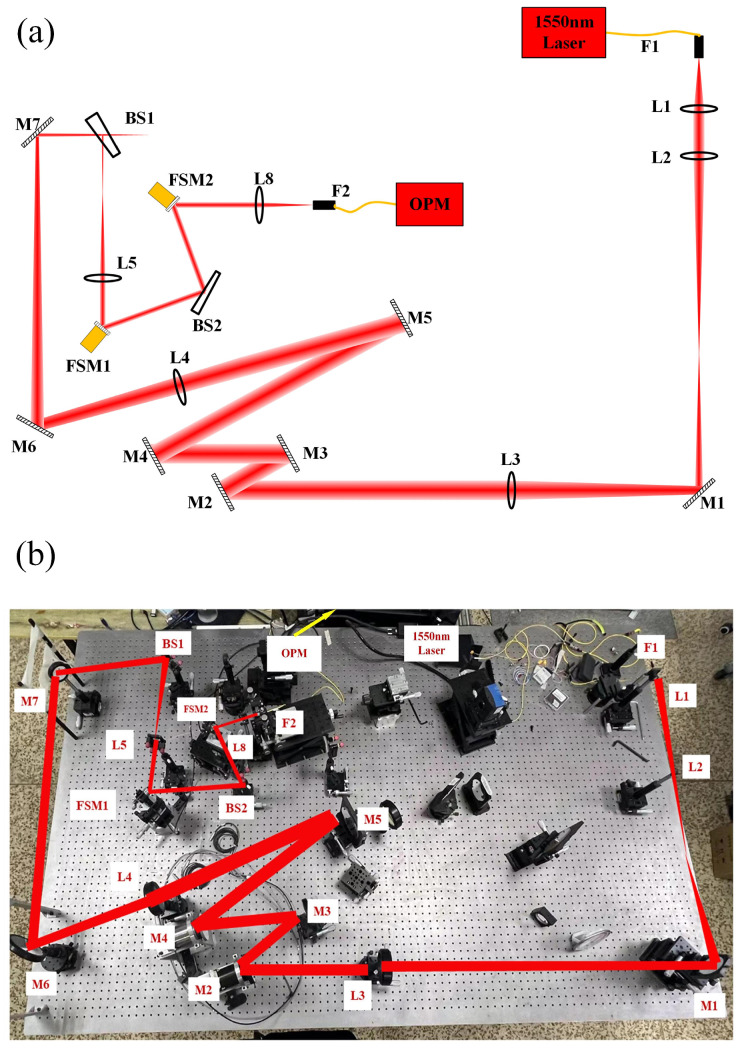
Experimental light path diagram: (**a**) experimental light path diagram; (**b**) experimental light path physical diagram.

The experimental schematic is shown in Figure 10, and the diagram of the experimental optical path is shown in Figure 15.

The beam is emitted by a 1550 nm laser light source (F1) and passes through the optical mirror group at the front end of the system (L1–L3, M1–M5). It then travels through a beam reduction system (M6, M7, L4, L5) to reduce the beam aperture before being reflected by the disturbed FSM (FSM1). Finally, the nutation FSM (FSM2) and fiber-coupling lens (L8) direct the beam into the SMF (F2) for subsequent power collection, parameter calculation, and feedback.

The SMF used in the experiment has a diameter of fiber core of 8.2 μm, NA number of 0.14, and a mode field diameter of 10.4 ± 0.5 μm. The coupling lens has an effective aperture of 10 mm and a focal length of 50 mm, and the execution frequency of the algorithm in the experiment is 1 kHz.

The FSM used for coupling is the S-331.2SL piezoelectric FSM manufactured by PI, featuring a mirror diameter of 25 mm, an open-loop resolution of 20 nrad, an open-loop deflection angle of 3.5 mrad, a closed-loop deflection angle of 2 mrad, and a repeat positioning accuracy of 0.06 μrad. Its no-load resonance frequency is 2.6 kHz. The disturbance FSM is the S-330.2SL voice coil FSM, also produced by PI.

### 3.1. Static Coupling Experiment of Parameter Self-Adjusting Algorithm

Firstly, a static coupling experiment was conducted to compare the performance of the parameter self-adjusting nutation coupling algorithm with that of the traditional fiber nutation algorithm under static conditions. During the experiment, the disturbance FSM1 remained stationary, while voltages were applied to the two axes of the coupling FSM2 to simulate static alignment deviations. The iteration started from this position and continued until the position of maximum coupling power was found.

Due to the linear relationship between the control voltage of the FSM and the resulting deflection angle, the initial static error and the set values of the initial nutation step length and radius were represented by voltage values in the experimental records, with the unit being volts (V).

The traditional fiber nutation algorithm1 used for comparison employed a nutation radius of r =0.05 V, a nutation step length of d =0.12 V, and a sampling point of n=6. The traditional fiber nutation algorithm2 employed a nutation radius of r =0.03 V, a nutation step length of d =0.15 V, and a sampling point of n=6. Nine different positions were selected for the initial alignment error: (0 V, 10 V), (2 V, 8 V), (3.5 V, 6.5 V), (3.5 V, 3.5 V), (5 V, 5 V), (6.5 V, 6.5 V), (6.5 V, 3.5 V), (8 V, 2 V), and (10 V, 0 V). The values of these positions covered the entire range of the FSM (0–10 V). The experimental results under different initial alignment errors are shown in Table 6. Steady accuracy is the root mean square (RMS) value of the power in the unit time after the power reaches the stability.

**Figure 16:**
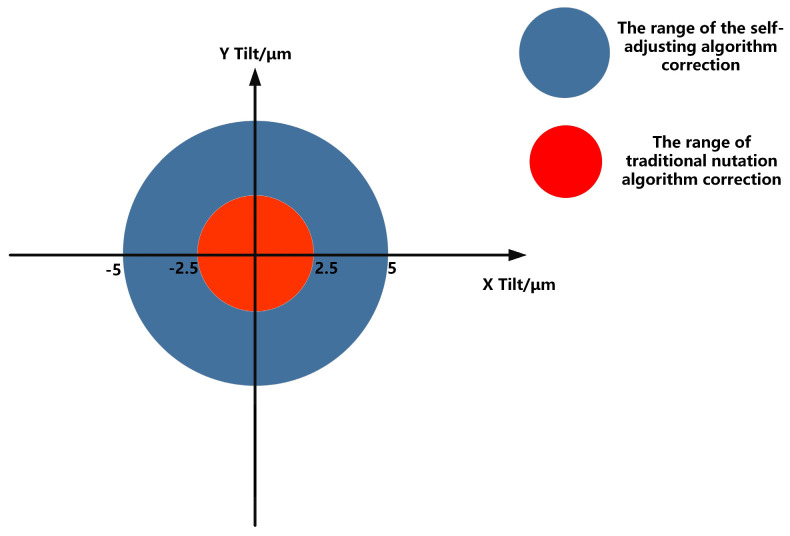
Algorithm calibration range comparison diagram.

Analysis of the results reveals that the parameter self-adjusting nutation coupling algorithm achieves coupling speeds approximately 20 times faster than the traditional fiber nutation algorithm. Moreover, the correction range for static deviations is significantly larger with the parameter self-adjusting nutation coupling algorithm, successfully coupling the Airy spot into the SMF core in all experimental groups 1 to 9, whereas the traditional algorithm only succeeds in control groups 3 to 7 with small static alignment errors. The correction range of the parameter self-adjusting nutation algorithm is about five times that of the traditional algorithm, as shown in Figure 16. Additionally, the steady-state accuracy of the parameter self-adjusting fiber nutation coupling algorithm is at least 0.3 dB higher than that of the traditional fiber nutation algorithm.

### 3.2. Dynamic Coupling Experiment of Parameter Self-Adjusting Algorithm

Based on the experiment in Section 3.1, an external single-axis sinusoidal disturbance was introduced, and the disturbance suppression bandwidth of the algorithm was measured. A signal generator was connected to the disturbance FSM1 to control it, producing sinusoidal disturbances with specific amplitudes and frequencies to introduce external disturbances. The power fluctuations detected by the optical power meter at the receiving end were compared to calculate the system’s disturbance suppression bandwidth.

This experiment was still compared with the traditional fiber nutation algorithm, with the introduced sinusoidal disturbance set to a PV of 100 μrad. The initial parameters for the traditional fiber nutation algorithm were the same as those in Section 3.1.

For the convenience of comparison, the power values and fluctuations detected by the optical power meter under the same frequency and uncorrected conditions, and after correction using both the parameter self-adjusting nutation coupling algorithm and the traditional fiber nutation coupling algorithm, are plotted on a single graph. The vertical coordinate represents the power value, and the horizontal coordinate represents the number of iterations.

**Figure 17:**
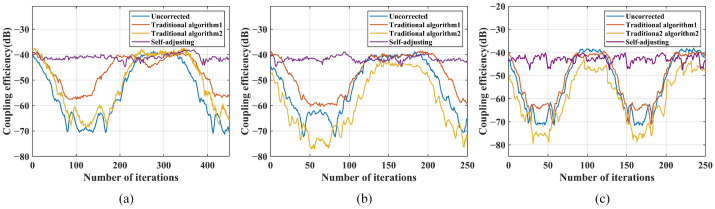
Comparison of coupling performance under 0.3 Hz, 0.5 Hz, and 0.8 Hz disturbance: (**a**) 0.3 Hz; (**b**) 0.5 Hz; (**c**) 0.8 Hz.

The comparison of coupling performance under disturbances of 0.3 Hz, 0.5 Hz, and 0.8 Hz is shown in Figure 17.

Under disturbance conditions of 0.3 Hz, both the parameter self-adjusting nutation coupling algorithm and the traditional nutation algorithm demonstrated suppression effects. Compared to the traditional nutation algorithm, the parameter self-adjusting nutation coupling algorithm increases the minimum coupling power by 12.42 dB and 24.38 dB, respectively, indicating that the correction effect of the parameter self-adjusting nutation coupling algorithm is better. Under 0.5 Hz disturbance conditions, the traditional fiber nutation algorithm2 (r =0.03 V, d =0.15 V, n =6) failed to suppress the disturbance, under 0.8 Hz disturbance conditions, the traditional fiber nutation algorithm1 (r =0.05 V, d =0.12 V, n =6) failed to suppress the disturbance, while the parameter self-adjusting fiber nutation coupling algorithm achieved a maximum coupling power of −39.5 dBm and a minimum coupling power of −47.0 dBm, still demonstrating effective correction.

**Figure 18:**
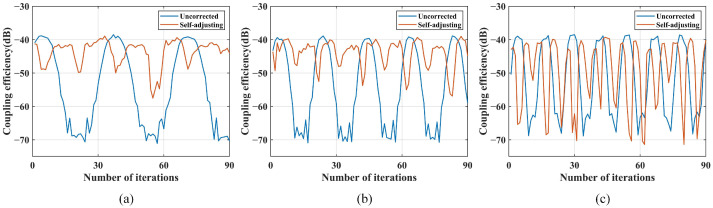
Comparison of coupling performance under 3.0 Hz, 5.0 Hz, and 8.0 Hz disturbance: (**a**) 3.0 Hz; (**b**) 5.0 Hz; (**c**) 8.0 Hz.

To test the upper limit of the suppression capability of the parameter self-adjusting fiber nutation coupling algorithm, the disturbance frequency is further increased. The coupling performance under disturbances of 3.0 Hz, 5.0 Hz, and 8.0 Hz is shown in Figure 18.

The coupling power range in the uncorrected state is approximately −39 dBm to −71 dBm. After applying the parameter self-adjusting fiber nutation coupling algorithm under a 3.0 Hz disturbance, the coupling power range from −39.3 dBm to −57.5 dBm, indicating effective correction. Under a 5.0 Hz disturbance, the coupling power range is −39.1 dBm to −58 dBm, still showing some correction. However, under an 8.0 Hz disturbance, the coupling power range shifts from −38.5 dBm to −73 dBm, indicating no correction effect. Therefore, the actual disturbance suppression bandwidth of the system using the parameter self-adjusting nutation coupling algorithm is 8.0 Hz.

### 3.3. Summary

The parameter self-adjusting nutation coupling algorithm offers significant advantages over traditional fiber nutation algorithms in terms of adjustment range, speed, and steady-state accuracy under static conditions. Compared to the traditional fiber nutation algorithm, it increases the disturbance suppression bandwidth by a factor of 10 and greatly enhances coupling stability. Therefore, under the current system conditions, the parameter self-adjusting SMF nutation coupling algorithm proves to be more practical.

## 4. Conclusions

This paper introduces a novel parameter self-adjusting SMF nutation coupling algorithm based on fuzzy control. Through comprehensive simulations and experiments, the algorithm’s performance in fiber nutation coupling systems has been evaluated. The study emphasizes the critical role of fuzzification, fuzzy inference, and defuzzification processes in meeting the specific needs of the algorithm. The results reveal that the proposed algorithm significantly enhances adjustment range, speed, and steady-state accuracy under static conditions, outperforming traditional nutation algorithms. Additionally, under dynamic conditions, it boosts disturbance suppression bandwidth by a factor of 10. Overall, the proposed algorithm effectively improves coupling performance, providing valuable insights and new directions for the advancement of fiber nutation algorithms.

## Figures and Tables

**Table 1:** Simulation parameters of fiber nutation algorithm.

Fixed Parameter	Parameter Value
Optical Maser Wavelength λ	1550 nm
Coupled Lens Equivalent Focal Length f	50 mm
Mode Field Diameter $\omega_{0}$	5.2 μm
Diameter D	10 mm

**Table 2:** Table of fuzzy rules.

Controls Parameter	*Pt*		*P*	
		*S*	*M*	*L*
*r*	*S*	*XL*	*M*	*VS*
	*L*	*L*	*M*	*S*
*d*	*S*	*XL*	*M*	*VS*
	*L*	*L*	*M*	*S*
*n*	*S*	*F*	*F*	*H*
	*L*	*F*	*H*	*H*

**Table 3:** Comparison of coupling performance without disturbance.

	Combined Parameter Selection				Consequence	*η*	*σ*	Iterations
		r /μm	d /μm	n				
Combination 1		0.25	0.60	4	Success	0.678	0.0030	98
Combination 2		0.2	0.50	6	Success	0.680	0.0026	114
Combination 3		0.15	0.40	4	Success	0.681	0.0016	138
Self-adjusting					Success	0.683	0.00010	82

**Table 4:** Parameters and results of fiber nutation algorithm.

Experimental Group	r	d	n	At 0.5 Hz			At 1.0 Hz		
				PV	RMS	*σ*	PV	RMS	*σ*
Combination 4	0.50	0.80	4	0.805	0.541	0.236	0.807	0.352	0.255
Combination 5	0.50	1.00	6	0.469	0.669	0.137	0.807	0.347	0.252
Combination 6	0.60	1.20	8	0.388	0.704	0.109	0.807	0.347	0.252
Combination 7	0.60	1.40	4	0.711	0.766	0.051	0.814	0.347	0.252
Combination 8	0.70	1.60	6	0.614	0.650	0.151	0.814	0.407	0.263
Combination 9	0.70	1.80	8	0.497	0.683	0.128	0.814	0.438	0.259
Combination 10	0.80	2.00	4	0.518	0.674	0.120	0.779	0.497	0.244
Combination 11	0.80	2.20	4	0.525	0.687	0.123	0.711	0.565	0.199
Self-adjusting				0.180	0.773	0.041	0.408	0.697	0.110

**Table 5:** Inhibition effect under different perturbations.

Parameter Combination	Self-Adjusting	4	5	6	7	8	9	10	11
Bandwidth/Hz	10	0.8	0.8	0.9	0.9	1.1	1.1	1.2	1.2

**Table 6:** Inhibition effect under different perturbations: a comparison between the traditional nutation 1, traditional nutation 2 and the parameter self-adjusting.

	Initial Position	Coupling Result			Number of Iterations/Times			Steady Accuracy/dBm		
		Self-Adjusting	Tradition1	Tradition2	Self-Adjusting	Tradition1	Tradition2	Self-Adjusting	Tradition1	Tradition2
1	(0 V, 10 V)	Success	Failure	Failure	14	None	None	−36.9	−54.5	−53.2
2	(2 V, 8 V)	Success	Failure	Failure	12	None	None	−36.8	−54.6	−53.8
3	(3.5 V, 6.5 V)	Success	Success	Success	8	144	132	−36.9	−37.5	−37.2
4	(3.5 V, 3.5 V)	Success	Success	Success	8	138	125	−37.0	−37.6	−37.4
5	(5 V, 5 V)	Success	Success	Success	3	68	49	−36.9	−37.7	−37.5
6	(6.5 V, 6.5 V)	Success	Success	Success	7	136	122	−36.8	−37.4	−37.2
7	(6.5 V, 3.5 V)	Success	Success	Success	8	154	141	−37.0	−37.6	−37.4
8	(8 V, 2 V)	Success	Failure	Failure	11	None	None	−36.7	−54.8	−53.6
9	(10 V, 0 V)	Success	Failure	Failure	14	None	None	−36.9	−55.2	−54.4

## Data Availability

The raw data supporting the conclusions of this article will be made available by the authors on request.

## References

[1] Kaushal H., Kaddoum G. (2017). Optical communication in space: Challenges and mitigation techniques. IEEE Commun. Surv. Tutor..

[2] Guiomar F.P., Fernandes M.A., Nascimento J.L., Rodrigues V., Monteiro P.P. (2022). Coherent free-space optical communications: Opportunities and challenges. J. Light. Technol..

[3] Kolev D.R., Carrasco-Casado A., Trinh P.V., Shiratama K., Ishola F., Kotake H., Nakazono J., Saito Y., Kunimori H., Kubooka T. (2023). Latest Developments in the Field of Optical Communications for Small Satellites and Beyond. J. Light. Technol..

[4] Liu Z., Gao S., Wu J., Chen Y., Ma L., Yu X., Wang X., Li R. (2024). Research on the NI-MLA Method for Enhancing the Spot Position Detection Accuracy of Quadrant Detectors Under Atmospheric Turbulence. Sensors.

[5] Velasco L., Ahmadian M., Ortiz L., Brito J.P., Pastor A., Rivas J.M., Barzegar S., Comellas J., Martin V., Ruiz M. (2024). Scenarios for Optical Encryption Using Quantum Keys. Sensors.

[6] Liang H., Yi Z., Ling H., Luo K. (2025). Modeling and Simulation of Inter-Satellite Laser Communication for Space-Based Gravitational Wave Detection. Sensors.

[7] Li Z., Pan Z., Li Y., Yang X., Geng C., Li X. (2023). Advanced root mean square propagation with the warm-up algorithm for fiber coupling. Opt. Express.

[8] Dikmelik Y., Davidson F.M. (2005). Fiber-coupling efficiency for free-space optical communication through atmospheric turbulence. Appl. Opt..

[9] Zhao X., Hou X., Zhu F., Li T., Sun J., Zhu R., Gao M., Yang Y., Chen W. (2019). Experimental verification of coherent tracking system based on fiber nutation. Opt. Express.

[10] Takenaka H., Toyoshima M., Takayama Y. (2012). Experimental verification of fiber-coupling efficiency for satellite-to-ground atmospheric laser downlinks. Opt. Express.

[11] Lv F., Liu Y., Gao S., Wu H., Guo F. (2023). Research on Bandwidth Improvement of Fine Tracking Control System in Space Laser Communication. Photonics.

[12] Swanson E.A., Bondurant R.S. (1990). Using fiber optics to simplify free-space lasercom systems. Proc. SPIE.

[13] Hu Q., Zhen L., Mao Y., Zhu S., Zhou X., Zhou G. (2020). Adaptive stochastic parallel gradient descent approach for efficient fiber coupling. Opt. Express.

[14] Bian Y., Li Y., Chen E., Li W., Hong X., Qiu J., Wu J. (2022). Free-space to single-mode fiber coupling efficiency with optical system aberration and fiber positioning error under atmospheric turbulence. J. Opt..

[15] Zhang L., Yu X., Zhao B., Wang T., Tong S. (2022). Method for 10 Gbps near-ground quasi-static free-space laser transmission by nutation mutual coupling. Opt. Express.

[16] Li Z., Pan Z., Li Y., Yang X., Li F., Geng C., Li X. (2024). Parameter-free fiber coupling method for inter-satellite laser communications based on Gaussian approximation. J. Opt. Commun. Netw..

[17] Pan Z., Li Z., Li Y., Huang G., Zou F., Pan L., Lin M., Li F., Geng C., Li X. (2025). Experimental demonstration of free-space optical communication under 2 km urban atmosphere using adaptive fiber coupling. Opt. Commun..

[18] Li B., Liu Y., Tong S., Zhang L., Yao H. (2018). Adaptive Single-Mode Fiber Coupling Method Based on Coarse-Fine Laser Nutation. IEEE Photonics J..

[19] Zhu S.W., Sheng L., Liu Y.K., Zhang Y., Gao S. (2019). Laser Nutation Coupling Algorithm for Single Mode Fiber Based on Energy Feedback. Chin. J. Lasers.

[20] Toyoshima M. (2006). Maximum fiber coupling efficiency and optimum beam size in the presence of random angular jitter for free-space laser systems and their applications. J. Opt. Soc. Am. A.

[21] Chen B., Liu X.P., Ge S.S., Lin C. (2012). Adaptive fuzzy control of a class of nonlinear systems by fuzzy approximation approach. IEEE Trans. Fuzzy Syst..

[22] Labiod S., Boucherit M.S., Guerra T.M. (2005). Adaptive fuzzy control of a class of MIMO nonlinear systems. Fuzzy Sets Syst..

[23] Wang J., Rad A., Chan P. (2001). Indirect adaptive fuzzy sliding mode control: Part I: Fuzzy switching. Fuzzy Sets Syst..

[24] Passino K.M., Yurkovich S. (1998). Fuzzy Control.

[25] Zhang H., Liu D. (2006). Fuzzy Modeling and Fuzzy Control.

[26] Cai X., Fu Z., Xie H., Xue J., Luo H., Ou N., Zhou G. (2022). Energy-Feedback Load Simulation Algorithm Based on Fuzzy Control. Appl. Sci..

[27] Chen Y.-T., Hsiu H., Chen R.-J., Chen P.-N. (2023). Stability of virtual reality haptic feedback incorporating fuzzy-passivity control. Meas. Control.

